# Symmetrical Dimethylarginine as a Diagnostic Parameter in Hermann's Tortoises (*Testudo hermanni*)

**DOI:** 10.3389/fvets.2022.824748

**Published:** 2022-02-22

**Authors:** Verena Lehmann, Barblin Altherr, Nikola Pantchev, Sabine Öfner, Yury Zablotski, Rachel Murphy, Michael Coyne, Petra Kölle

**Affiliations:** ^1^Clinic of Small Animal Medicine, Ludwig Maximilian University of Munich, Munich, Germany; ^2^IDEXX Med Labor GmbH, Kornwestheim, Germany; ^3^Reptile Rescue Center Munich, Munich, Germany; ^4^IDEXX Laboratories, Inc., Westbrook, ME, United States

**Keywords:** kidney disease, renal disease, chelonian, reptile, uric acid

## Abstract

**Background:**

Despite improvements in habitational conditions, kidney disease is relatively common in tortoises.

**Objectives:**

Purpose of this study was the establishment of Symmetrical dimethylarginine (SDMA) reference values for clinically healthy Hermann's Tortoises.

**Animals:**

Clinically healthy Hermann's Tortoises (*n* = 131) were included in the period from October 2017 to September 2019.

**Methods:**

Creatinine and other biomarkers were tested at IDEXX Laboratories, Germany using residual blood samples from Hermann's tortoises. SDMA was measured with the IDEXX test and verified by liquid chromatography-mass spectrometry at IDEXX Laboratories, USA.

**Results:**

SDMA values ranged from 1 to 21 μg/dl (*n* = 131) for the IDEXX SDMA Test and SDMA values ranged from 1 to 17 μg/dl (*n* = 82) for LC-MS. For the comparison of the two measuring systems, the following results were obtained *R*^2^ = 0.75 (*p* < 0.001).

**Conclusion and Clinical Importance:**

SDMA can be measured in Hermann's Tortoises and the reference values range in clinically healthy animals is comparable to that of dogs and cats.

## Introduction

With the growing numbers in reptiles kept as pets, veterinarians will be increasingly challenged with the treatment of these exotic animals. Among the most popular reptiles kept by private owners in Germany are *Testudo* spp., especially the Hermann's Tortoise (*Testudo hermanni*) (1–3), comprising two subspecies, *Testudo hermanni hermanni* and *Testudo hermanni boettgeri*. Tortoises are particularly prone to kidney diseases (4). At necropsy, it was found that 64.30% of the animals showed kidney disease as a primary or secondary cause of death (5–7). It was revealed that the Greek tortoise and the Spur-thighed tortoise (*Testudo graeca*) have a higher incidence of renal disease in clinical examination as well as in necropsy compared to the Afghan tortoise (*Agrionemys horsfieldii*) and the Marginated tortoise (*Testudo marginata*) (5). Non-specific clinicals signs often leads to diagnosis of reduced kidney function at more advanced stages of a chronic disease, resulting a negative outlook for survivability (2, 8, 9). Furthermore, with few biomarkers specifically relating to the kidney, such as dietary influenced uric acid, detection of decline in renal function is more challenging (10). Feeding tortoises mainly with protein-rich food usually causes an increase in uric acid levels (9). Continual feeding of protein-rich diets may cause serious diseases, such as kidney and liver disease, bladder stones, and gout (9). The availability of an earlier diagnosis with a specific and targeted diagnostic test could positively affect prevention and a better long-term management for reptiles (8).

Even in humans studies have also used SDMA as a research marker for indirect determination of glomerular filtration rate and thus for the assessment of kidney function (11, 12). In 2015, the new endogenous biomarker SDMA was established for both dogs and cats to identify renal disorders (13–15). As a result of posttranslational processes in nucleated cells, methyl residues are attached to arginine residues by the enzyme protein arginine methyltransferase 5 (PRMT5) and, after proteolysis, SDMA and asymmetric dimethylargine are released into the circulatory system (16). In contrast to ADMA, SDMA is not degraded enzymatically but mainly excreted *via* the kidneys. Therefore, it is a suitable parameter for the early detection of kidney disease (17, 18). In contrast to serum creatinine, SDMA is much less affected by non-renal influence like muscle mass and diet (13, 14). Moreover, SDMA often increases earlier in the event of chronic kidney disease than creatinine, and can therefore be used as an early indicator of disease (13, 15, 17).

The purpose of the present study is to evaluate the measurability of SDMA in reptiles and to establish reference values of SDMA in clinically healthy Hermann's Tortoises.

## Materials and Methods

### Study Design

The data were collected from October 2017 to September 2019. Only clinically healthy tortoises that weighed more than 0.45 kg were included. A total of 131 tortoises (98 male and 33 female specimens) were examined. The average weight was 1.1 ± 0.60 kg. The majority of animals belonged to the Reptile Rescue Center Munich, Germany, which shelters abandoned animals. A total of 14 tortoises were contributed by the Zoo Neumuenster, Germany and an additional seven tortoises of two private owners. All tortoises were kept in outdoor enclosures or terrariums with species-appropriate husbandry conditions. Hibernation was performed from end of October to March.

Due to the hygiene protocol at the Reptile Rescue Center Munich every new tortoise must undergo blood tests on herpesvirus infection and routine biochemical profiles are also performed. The present study used these residual blood samples for measurement of serum biomarkers, of renal functions [UA, urea, calcium, phosphate, alanine–aminotransferase (ALT), aspartate–aminotransferase (AST)], and SDMA. With only a few exceptions, blood was drawn from the dorsal tail vein (Vena coccygealis dorsalis) of the tortoises without sedation, which is considered an established technique for blood collection (2, 9). The recommended maximum amount of whole blood is 0.80% of the bodyweight of an animal in good general condition (2, 19, 20). In this study, this was equivalent to an average amount of 1.5–2.5 ml. Thereafter, the sample was centrifuged at 3,000 rpm (Hettich Eba 3S) and the serum was pipetted into a sample tube for sending to IDEXX. Within the laboratory control of the Reptile Rescue Center, Munich, alkaline phosphatase [ALP], glutamate-dehydrogenase [GLDH], ALT, AST, creatine kinase [CK], total protein [TP], Blood urea nitrogen (BUN), uric acid [UA], inorganic phosphorus [P], total calcium [Ca], sodium [Na], and potassium [K] were analyzed with a Beckman Coulter Olympus AU5800 chemistry analyzer (Beckman Coulter GmbH, 47807 Krefeld, Germany).

For the analysis of SDMA in serum and plasma, a novel high throughput homogenous competitive immunoassay by IDEXX Laboratory was used, which based on a glucose-6-phosphate dehydrogenase conjugate and anti-SDMA monoclonal antibodies.

To verify the immunoassay results, samples were retested with LC-MS at IDEXX Laboratories, Westbrook, ME, USA. A total amount of 100 μl of the sample were used for the LC-MS method and 17 μl for the IDEXX SDMA test. The SDMA concentration was measured in each individual tortoise sample using LC-MS/MS following previously described methods (17).

### Statistics

Statistical analyses were performed using R 4.0.3 (2020-10-10, R Foundation for Statistical Computing, Vienna, Austria) (21). Results with a *P* < 0.05 were considered statistically significant, while results with a *P*-values between 0.1 and 0.05 were considered suggestive. The normality of SDMA and UA distribution was assessed both with the Shapiro–Wilk normality tests and visually using Quantile–Quantile plots. Due to a non-normal distribution, data were further analyzed using a non-parametric two sample Mann–Whitney test (22). Due to its reliability in empirical work, the Spearman correlation was selected to determine correlations between SDMA and UA and Ca/P ratio. For further analysis of the SDMA value, the total number of tortoises was divided into two groups based on a cut-off value of UA (>5.20 mg/dl) (19).

Verification by LC-MS measurement was performed on 82 tortoises. Due to repeated measures on several animals and highly unbalanced design, we initially fitted a linear mixed-effects-model (estimated using REML and nloptwrap optimizer) to predict “sdma_immunoassay_mg_dl_0_14” with “sdma_lc_ms_messung” with a random effect of the “animal.” However, due to (1) singular fit, (2) zero explained variance by the random effect and (3) due to the absence of significant difference between variances of a model with and a model without random effect (estimated by AIC), we fitted a simple linear model. The following model assumptions were checked: (1) the normality of residuals was checked by the Shapiro-Wilk normality test, (2) the independence of residuals (autocorrelation of error terms) was checked by the Durbin–Watson-Test, the heteroscedasticity (constancy of error variance) was checked with Breusch–Pagan test. Due to (1) the violation of some assumptions and (2) the inability to relax those assumptions by data transformation (log- and square root transformations were tested), we fitted a robust linear regression with the Design Adaptive Scale estimate as proposed in Koller and Stahel (23). The choice of the model resulted in the significant improvement of model fit from the *R*^2^ of 0.27 for the simple linear regression to the *R*^2^ of 0.75 for the robust regression.

## Results

SDMA values showed a range from 1 to 21 μg/dl (95 % CI: 3-16 μg/dl, *n* = 131; Table 1).

**Table 1:** SDMA levels of female and male Hermann's Tortoises (*n* = 131).

**SDMA (μg/dl)**	**Mean**	**Minimum**	**Maximum**	**Q 0.025**	**Q 0.975**
Total amount	9	1	21	3	16
(*n* = 131)					
Females (*n* = 33)	10	3	21	9	12
Males (*n* = 98)	8	1	17	7	9

**Figure 1:**
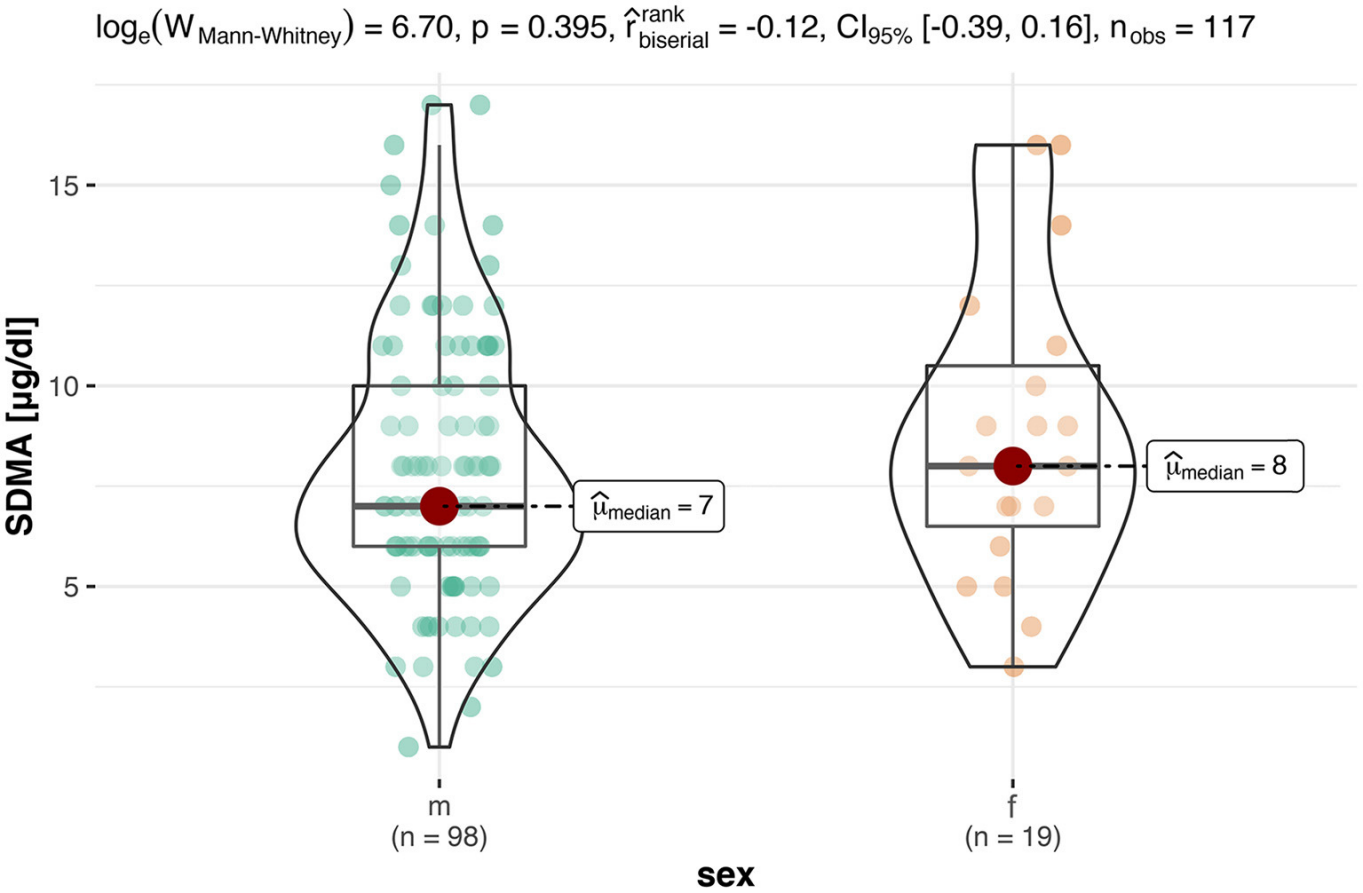
This box-violin-plot shows the distribution of SDMA levels in both sexes without animals from Neumeunster Zoo. The colored dots represent each individual data point, while the shape of the bean plot represents its density. In addition, the minimum and maximum values as well as the median are shown as boxplots.

Since 14 of the 33 females belonged to the same population, it was investigated whether this had an effect on the analysis of the SDMA values. For this purpose, this group was not included in the calculation of the statistical differences with regard to sex for the final analysis. Consequently, the results showed that there were no significant differences (*p* = 0.40) in sex for SDMA, which would be in agreement with other studies in which no difference by sex was found (24) (Figure 1).

**Figure 2:**
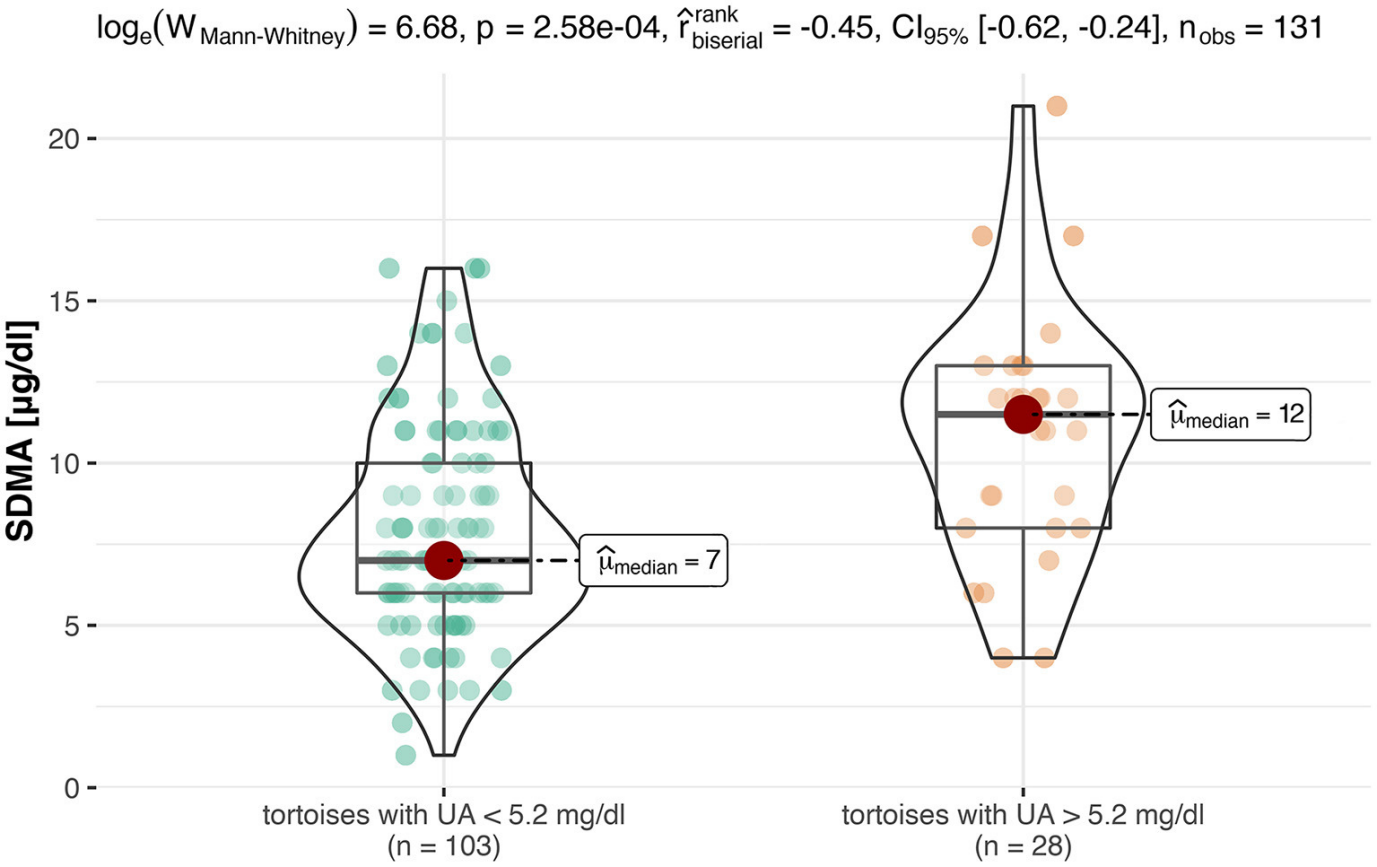
This box-violin-plot shows the distribution of SDMA values after creating two groups based on UA values. The colored dots represent each individual data point, while the shape of the bean plot represents its density. In addition, the minimum and maximum values as well as the median are shown as boxplots.

**Figure 3:**
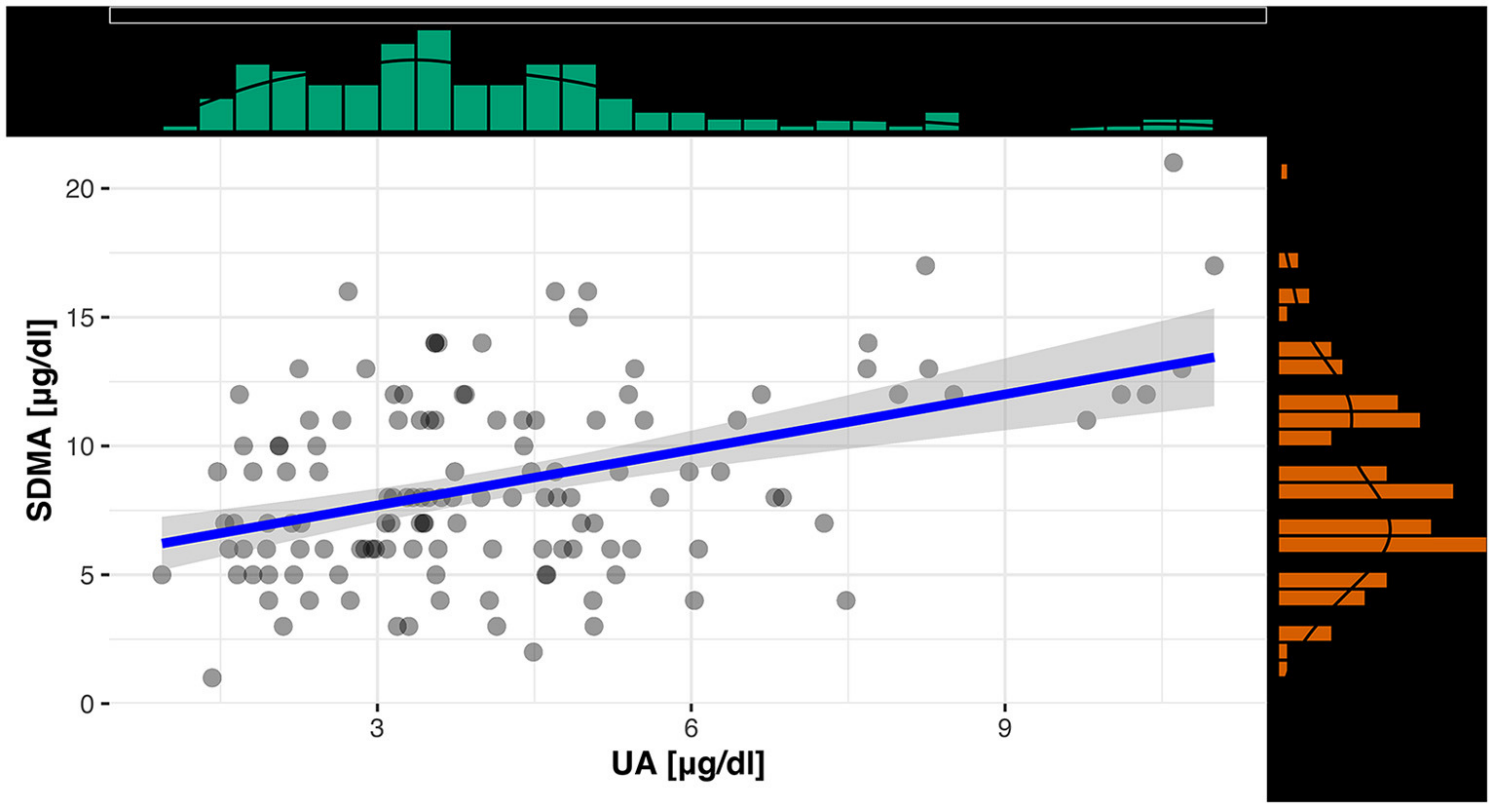
Scatterplot with regression line and marginal distribution: correlation between SDMA and UA.

**Figure 4:**
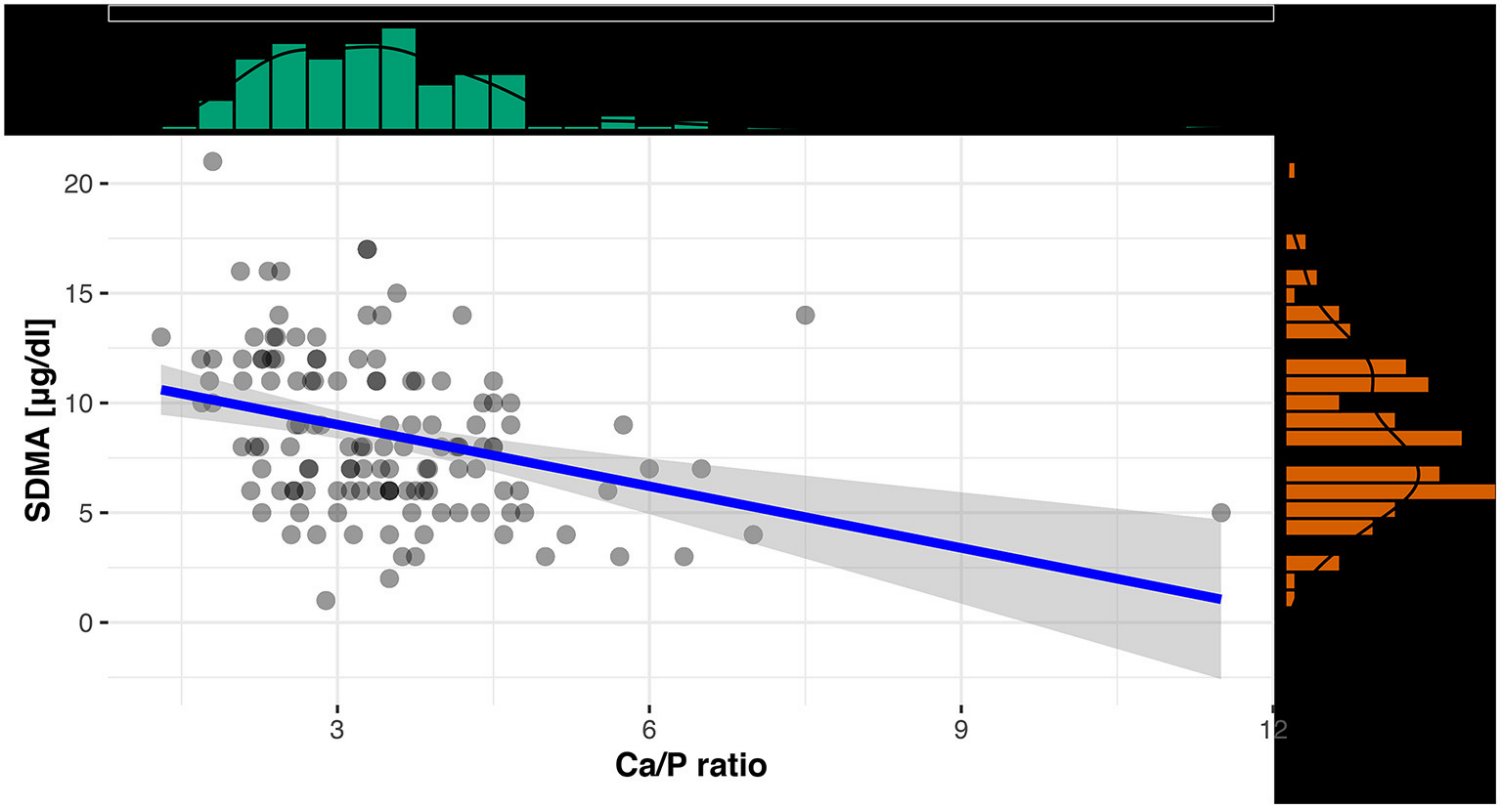
Scatterplot with regression line and marginal distribution: correlation between SDMA and Ca/P ratio.

For an evaluation of a potential correlation between SDMA and the commonly used kidney parameter UA (for reptiles), the data of the clinically healthy animals were divided into two groups. The differentiation between tortoises with normal UA concentrations and tortoises with elevated UA concentrations was based on the reference interval of 0-5.2 mg/dl UA (19). Out of 131 tortoises, 103 animals showed normal UA levels with SDMA 95% CI: 3-16 μg/dl, while 28 animals had elevated levels with SDMA 95 % CI: 4-18 μg/dl (Figure 2). Mann–Whitney *U-*Test confirmed it as a significant difference (*p* < 0.001). Spearman correlation coefficient confirmed a correlation between SDMA and UA of ρ_s_ = 0.32 (*p* < 0.001) (Figure 3) and between SDMA and Ca/P ratio of ρ_s_ = −0.39 (*p* < 0.001) (Figure 4). No significant correlation was found with other parameters measured in this study (urea, aspartate aminotransferase, and phosphate).

**Figure 5:**
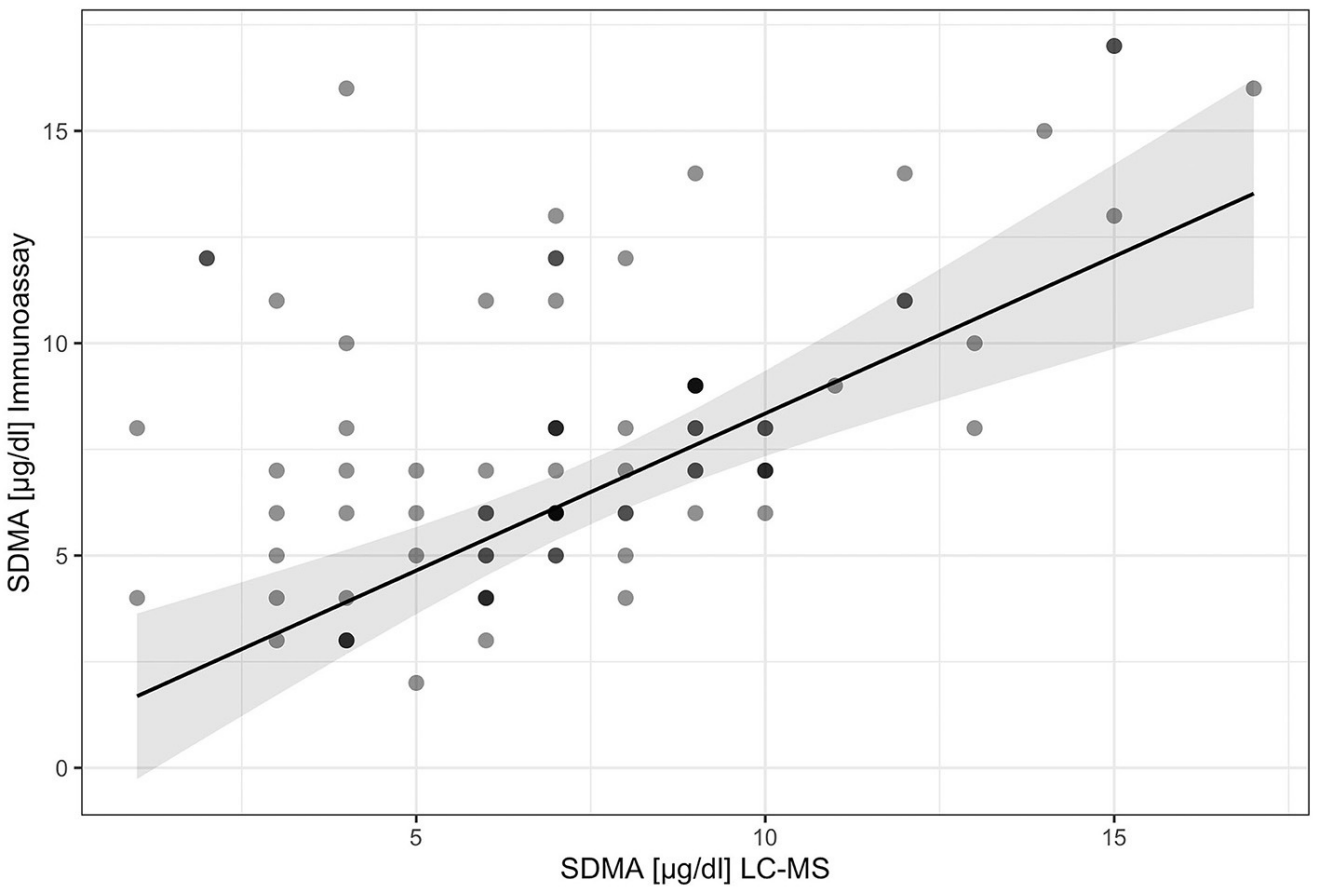
Scatterplot with regression line: comparison of the two measurement methods for SDMA.

The results from the LC-MS measurement show a high correlation between the gold standard method and the IDEXX SDMA test with a value of *R*^2^ = 0.75 (*p* < 0.001; Figure 5).

## Discussion

Several studies in dogs and cats demonstrated that SDMA, unlike creatinine, is rarly influenced by extra kidney factors like lean body mass or diet (17, 24, 25) and can be used as an early diagnostic parameter for the indication of a kidney disease in the future (13, 14, 17). To date, no study has been performed on the measurability and applicability of SDMA in tortoises or other reptile groups. It can be assumed that SDMA in reptiles is also unaffected by the diet, which needs to be investigated further by future studies.

In tortoises, general and thorough examination has certain limitations due to the specific physiology and anatomy compared to common pets such as dogs and cats. Reptiles generally show few and very unspecific symptoms. Due to the shell of the tortoise, a comparable examination for abdominal palpation is only possible to a very limited extent. Therefore, further diagnostics, such as laboratory tests play a particularly important role (26). In tortoises, blood samples can be taken without sedation at several locations, such as the dorsal tail vein (*Vena coccygealis dorsalis*) or the supravertebral venous plexus (*Venus subcarapacialis*) (2). Through the analysis of the blood samples, relevant blood parameters can be determined which could fill the gap left by the limitations of the general examination, allowing a thorough and timely diagnosis to be achieved. However, it is important to note that reference ranges may vary greatly between different tortoise species (26, 27) and also depending on the site of blood collection (28).

Depending on the tortoise species, the end products of protein, nitrogen, and purine metabolites are excreted in different ways. This makes a significant difference in diagnosing kidney disease. As Hermann's Tortoises are uricotelic, UA can be used as a diagnostic parameter in contrast to mammals for which urea and creatinine are the most important parameters for the diagnosis of kidney disease (29). However, UA is not an ideal kidney function parameter for reptiles, as several studies confirmed that this parameter increases at a late stage of kidney disease. Therefore, it is not considered appropriate for an early treatment of potential kidney disease (9, 30). Given the viability of SDMA as an early indicator, the present study also evaluated a potential correlation between SDMA and UA.

A number of studies with tortoises indicated an influence of a protein-rich diet on kidney biomarkers. The study by Kölle (5) showed that the diet of European tortoises has a significant influence on UA. After feeding a diet with high protein content, UA increased significantly within 24 h and decreased gradually within 7-11 days. Thus, the UA level has only limited reliability for the diagnosis of kidney diseases in tortoises (5). Another study in snakes (*n* = 10) also confirmed that UA values fluctuated by the time point of feeding. Increased levels were observed at day 2-3 which are comparable to those of animals suffering from kidney diseases or gout (31).

In contrast to the increase of UA at a late stage of kidney disease, the calcium/phosphate ratio already changes at an earlier stage (9). However, this ratio is also influenced by non-kidney factors. In female reptiles, calcium increases during vitellogenesis, especially after hibernation, as there are increased requirements for yolk formation. In comparison, male tortoises have low calcium levels (19, 32, 33). The study of Andreani et al. (19) also indicated that sex and season have a major impact on several blood parameters in *Testudo hermanni*. The study was based on 34 animals (14 males, 20 females), some of which were tested in September and July of the following year. Andreani et al. (19) also detected that female tortoises had a lower concentration for UA and a higher concentration for calcium compared to male tortoises while the concentration of UA was higher in July compared to September for a mixed-sex group. All animals in this study were raised in captivity and therefore provide reliable information regarding age. Furthermore, the husbandry was under natural sunlight and the animals had the same plants available as nutrition. Physical examination was carried out before each blood sample was taken, which included assessment of eyes, nares, oral cavity, skin, and carapace. Due to the comparability of similar model type of analyzer, this study was suitable as a reference source for the UA values in the current study, as the study of Andreani et al. (19) used an Olympus AU5400 automated biochemical analyzer to measure the samples. Therefore, a cut-off value (>5.20 mg/dl) for the evaluation of the UA value was set, and at which point UA values are considered to be elevated. Another recent study surfaced similar results regarding the influence of sex and seasonality on most biochemical parameters. The study design consisted of a total of 256 samples (148 males, 108 females), which were assigned to the three different seasons (spring, summer, autumn) based on the date of sampling. In contrast to the study by Adreani et al. (19), the females showed an increase in UA value in autumn compared to summer. However, male tortoises were also shown to have higher UA values (27). Both studies indicate that it is particularly difficult to establish reference values in reptiles compared to endothermic animals, as not only the species but also the seasonality and the associated physiology are decisive. Therefore, future studies need to investigate whether SDMA is affected by collection at different times of the year.

The present study also analyzed the influence of the sex on SDMA. Results of the full study population indicated that females allegedly had significantly higher SDMA levels than male tortoises. A possible explanation is that the specific group of females (*n* = 14) in this study were kept together and probably all specimens showed elevated UA and SDMA levels due to keeping conditions. However, in other studies (5), it could be shown that males are more susceptible to kidney disease which should result in higher average SDMA levels in the male population.

Seasonal influences were not considered in the present study. Many blood parameters are known to show seasonal variations, i.e., UA is higher in spring (32). As seasonality does not apply to cats and dogs, no clear conclusion about a potential influence of the seasons to SDMA can be drawn yet.

In addition to the blood samples of the 131 tortoises used in the study, 11 samples were available from third-party veterinarians and were excluded from the study due to unclear health conditions. Samples were sent to IDEXX for routine diagnosis mostly. For all cases in which enough residual blood from the sample was available, IDEXX tested these additionally for SDMA to extend the available data for the measurability and applicability of SDMA in Hermann's Tortoises. From that additional number of Hermann's Tortoises, four specimens had high SDMA levels (>20 μg/dl) in combination with elevated UA levels (>7 mg/dl). Two of these samples were sent in because of anorexia, for the other two, no additional information on health status had been provided. Contrary to the elevated SDMA and UA findings, one sample showed increased UA (7.33 mg/dl) but low SDMA (3 μg/dl) levels. Several sources in literature confirmed that such a finding could be explained by dehydration as this would lead to increased UA levels in reptiles (34). Furthermore, a protein-rich nutrition could not be entirely excluded due to a lack of the patients' history.

The results of this study revealed that SDMA can be measured in blood samples of tortoises and that reference values could be generated. A potential limitation of this study is the lack of diversity in the ownership of the study population as over 85% of the animals were provided by the Reptile Rescue Center Munich. Especially because those tortoises had been kept there for an extended duration and had been exposed to similar habitat conditions and nutrition. As no diseased tortoises or wild populations were included in this study, this aspect should be further investigated in future research. Additional research is required to determine whether a correlation between seasonal influences on SDMA in tortoises can be detected as in the case for UA (27).

The purpose of this study was to evaluate the measurability of SDMA and establish reference values for the Hermann's Tortoise by analyzing residual blood samples of clinically healthy animals. To quantify SDMA in serum samples a high-throughput immunoassay was used (35). The LC-MS analysis, although accurate and considered the gold standard for the measurement of SDMA in blood samples, is a costly and time-consuming test. Therefore, it is not included to the routine laboratory minimum data base for sick and healthy pets. The IDEXX SDMA assay was validated for dogs and cats, using healthy animals and populations with kidney disease, but to date data for reptiles were missing. As a final verification of the SDMA values, 70% of the samples were retested using the LC-MS method. Since only residual blood samples were used in the study, ~30% of the samples did not have enough residual material for another analysis. The LC-MS method is considered the gold standard for measuring SDMA. Studies in dogs and cats have confirmed that the IDEXX SDMA Test is superior to other tests in direct comparison to the gold standard (36). This technique is particularly suitable for small biomarkers, such as SDMA, which has a little immunogenic effect due to its size (35). This assay correlates in a best fit linear model with a slope of 1.06, an intercept of 0.34 with *R*^2^ = 0.99 with the measurement by liquid chromatography-mass spectrometry, considered as the gold standard (36). Compared to the LC-MS, within-run precision was less than or equal to a 7% coefficient of variability (CV) in the range of 10–20 μg/dL, and the total precision was ≤ 10% CV (35). Another study was able to show that in the range relevant for the clinic for SDMA (10–45 μg/dl), the bias was 1-2 μg/dl for the IDEXX SDMA test, while the DLD SDMA Elisa for human samples had a considerably higher bias of 8-17 μg/dl for the same range. This study additionally revealed a total CV of 2.3% for the IDEXX test in samples with high SDMA concentration, whereas the human SDMA ELISA showed a CV of 28.2% (36). The IDEXX SDMA test defined a reference interval of 0-14 μg/dl for dogs and cats based on the transferability of SDMA values from LC-MS of healthy individuals, taking into account mean bias, method precision and integer rounding of the original data (35). Validated data already exists for IDEXX SDMA Test for cats, dogs, horses and rats, which were compared with LC-MS (37).

Since no studies have yet been performed on tortoises or other reptiles in SDMA, it is not yet clear whether the synthesis and metabolism are similar to those in mammals. This could be a possible explanation for the lower coefficient (*R*^2^ = 0.75) of determination compared to dogs and cats (*R* = 0.99). Another hypothesis is that there are other derivatives that are not found in the mammal. Another study conducted on draft horses yielded similar results with a strong correlation (*R* = 0.74, *p* < 0.001) between LC-MS and the immunoassay (38).

In summary, SDMA may be a valuable tool for early diagnosis of a decline kidney function in tortoises and may also become a constituent of laboratory tests for pre-purchase testing.

## Data Availability Statement

The raw data supporting the conclusions of this article will be made available by the authors, without undue reservation.

## Ethics Statement

The present study was approved by the Animal Protection and Ethics Council of the Veterinary Faculty of the Ludwig-Maximilians-University on 09/25/17 with reference number 90 05 07 17. Written informed consent for participation was not obtained from the owners because samples were not taken specifically for the study, but were taken as part of medical examinations and the remains were used for the study.

## Author Contributions

VL: study design, data collection, and preparation of the manuscript. BA and NP: study design, critical revision of manuscript, and measurements of the samples in Germany. SÖ: study design, data collection, and critical revision of manuscript. YZ: statistical analysis. RM and MC: measurements of the sample in USA and critical revision of manuscript. PK: study design and critical revision of manuscript. All authors contributed to the article and approved the submitted version.

## Conflict of Interest

BA, NP, RM, and MC were employed by company IDEXX. The remaining authors declare that the research was conducted in the absence of any commercial or financial relationships that could be construed as a potential conflict of interest.

## Publisher's Note

All claims expressed in this article are solely those of the authors and do not necessarily represent those of their affiliated organizations, or those of the publisher, the editors and the reviewers. Any product that may be evaluated in this article, or claim that may be made by its manufacturer, is not guaranteed or endorsed by the publisher.
